# Challenges in Implementing the Local Node Infrastructure for a National Federated Machine Learning Network in Radiology

**DOI:** 10.3390/healthcare11172377

**Published:** 2023-08-23

**Authors:** Paul-Philipp Jacobs, Constantin Ehrengut, Andreas Michael Bucher, Tobias Penzkofer, Mathias Lukas, Jens Kleesiek, Timm Denecke

**Affiliations:** 1 Department of Diagnostic and Interventional Radiology, University of Leipzig, 04109 Leipzig, Germany; 2 Department of Diagnostic and Interventional Radiology, Johann-Wolfgang-v.-Goethe-Universität, 60629 Frankfurt, Germany; 3 Department of Radiology, Campus Virchow-Klinikum, Charité—Universitätsmedizin Berlin, 10117 Berlin, Germany; 4 Institute for Artificial Intelligence in Medicine, University Hospital Essen (AöR), 45131 Essen, Germany; 5 Medical Faculty, University of Duisburg-Essen, 45122 Essen, Germany

**Keywords:** federated machine learning, network, infrastructure, medical imaging, radiology, RACOON

## Abstract

Data-driven machine learning in medical research and diagnostics needs large-scale datasets curated by clinical experts. The generation of large datasets can be challenging in terms of resource consumption and time effort, while generalizability and validation of the developed models significantly benefit from variety in data sources. Training algorithms on smaller decentralized datasets through federated learning can reduce effort, but require the implementation of a specific and ambitious infrastructure to share data, algorithms and computing time. Additionally, it offers the opportunity of maintaining and keeping the data locally. Thus, data safety issues can be avoided because patient data must not be shared. Machine learning models are trained on local data by sharing the model and through an established network. In addition to commercial applications, there are also numerous academic and customized implementations of network infrastructures available. The configuration of these networks primarily differs, yet adheres to a standard framework composed of fundamental components. In this technical note, we propose basic infrastructure requirements for data governance, data science workflows, and local node set-up, and report on the advantages and experienced pitfalls in implementing the local infrastructure with the German Radiological Cooperative Network initiative as the use case example. We show how the infrastructure can be built upon some base components to reflect the needs of a federated learning network and how they can be implemented considering both local and global network requirements. After analyzing the deployment process in different settings and scenarios, we recommend integrating the local node into an existing clinical IT infrastructure. This approach offers benefits in terms of maintenance and deployment effort compared to external integration in a separate environment (e.g., the radiology department). This proposed groundwork can be taken as an exemplary development guideline for future applications of federated learning networks in clinical and scientific environments.

## 1. Introduction

In recent years, the application of machine learning and artificial intelligence made its way into various topics of medical research and diagnostics [1,2]. Automated analysis of radiological images should especially be highlighted in this context [3,4,5,6]. Such data-driven approaches usually benefit from large-scale high-quality curated datasets [7,8], which are time- and resource-consuming to generate. Federated learning is a technique where algorithms can be trained decentralized on smaller datasets distributed across multiple sites, thus lowering the effort of data curation by each contributing party and helping solve data privacy problems [8,9]. One of the most significant advantages of federated learning in medical and radiology research is its ability to address data privacy concerns. In traditional machine learning methods, centralized data repositories pose a substantial risk to patient privacy, as sensitive medical information could be compromised in the event of a security breach [10]. Federated learning, on the other hand, allows data to remain localized on individual devices, ensuring that patient data stay within the secure confines of healthcare institutions [8,11,12,13]. It enables researchers to access a more diverse and extensive dataset without physically aggregating the data. In medical and radiology research, access to a diverse range of patient demographics, medical conditions and imaging modalities is crucial for building robust and generalizable models [14,15,16]. In this research field, ethical considerations are paramount when using patient data for research purposes. Federated learning adheres to ethical principles by minimizing data exposure, as the model parameters are the only pieces of information exchanged between the central server and the local devices. This approach ensures that individual patient data remain confidential while still contributing to the collective knowledge base for the benefit of all patients.

However, an infrastructure for sharing data and algorithms in a well-orchestrated and secure manner, in line with the country-specific data security policies is required. The establishment of such a configuration necessitates the coordination between locally implemented data science and processing workflow, as well as the interaction among the nodes involved through the network infrastructure. This can be accomplished through a standardized set of fundamental building blocks that have been previously implemented in both commercial and academic environments [17,18].

An example is the German Radiological Cooperative Network (RACOON) initiative [19,20,21,22], aimed to connect the radiology departments of all university hospitals in Germany to provide easy access to a large amount of curated data for multicentric studies. This kind of infrastructure can be used for federated data analysis and training of machine learning algorithms in medical diagnostics and research [6,18,20].

For this purpose, a dedicated setup of hardware and user services needs to be established at each participating site, comprising data curation and reporting workflows, as well as an interface for data transfer to external nodes.

In this technical note, we compare different site-specific setup realizations and discuss the advantages and pitfalls experienced during participation in the RACOON project. We present an analysis of generalized infrastructure requirements and compare differences in possible set-ups.

## 2. Materials and Methods

### 2.1. Data Governance and Data Sharing

Data governance is critical when dealing with medical data. Access to medical data needs to be carefully controlled and data must be protected against unauthorized access or misuse. Data governance policies and procedures must be established before building an infrastructure for federated learning. This should include the identification of sensitive data and the development of policies for data access, data storage and data sharing. The roles and responsibilities for data management, data security and data privacy should also be defined within governance policies.

Both anonymization and pseudonymization techniques play pivotal roles in ensuring the privacy and security of medical radiological data during sharing. It is essential to strike a balance between maintaining data utility for research and analysis while safeguarding patient privacy. The process of anonymization or pseudonymization should not compromise the utility of the data for research and clinical purposes. Sufficient contextual information should be preserved to maintain the data’s value without revealing identifiable details.

One way to handle the issues of data protection is to provide a Joint Controllership Agreement (JCA). The JCA provides information and rules on data handling, including de-identification and data sharing.

De-identification is a crucial step in ensuring the privacy and security of sensitive data when using federated learning. This includes the process of removing or obscuring identifying information from datasets to protect the privacy of individuals whose data are included. To ensure an effective process and adequate protection of sensitive data, some requirements should be considered. Depending on the jurisdiction and type of data involved, there may be legal and regulatory requirements governing the de-identification of data. These requirements should be carefully considered and incorporated into the de-identification process to ensure compliance with applicable laws and regulations. There are several techniques that can be used to de-identify data, including pseudonymization and anonymization. The appropriate technique depends on the specific use case and the nature of the data involved. The technique chosen must effectively remove or obscure identifying information. While anonymization is always the preferred method from a data protection perspective, it often drastically lowers the utility of the data for analysis, modeling and quality assurance. Strong or reliable pseudonymization, where the external recipient of the data never has access to the required keys for the re-identification, is therefore usually the most practical method. In the case of the RACOON project, we chose strong pseudonomization where the patient data are obscured through a hashing algorithm within the project’s own data management system. The keys for re-identification remain on the local servers and are never shared with the project partners or any third party.

When sharing data, it is important to document the data model and associate metadata to ensure that it can be effectively used and interpreted by other parties. This may include providing documentation on the structure, data definitions and any relevant metadata helping other parties understand the data. In the context of medical data, the DICOM (Digital Imaging and Communications in Medicine) standard is a widely used and flexible format to store and share data [23]. This data container format can store a wide range of different information such as image and text data and even machine learning models. The NIfTI (Neuroimaging Informatics Technology Initiative) format is another popular file format for sharing medical imaging data [24]. It primarily deals with three-dimensional imaging data, enabling the exchange of MRI, fMRI, CT data and data from various other modalities. When sharing NifTI data, pseudonymization techniques are often applied, which involves replacing direct identifiers with pseudonyms. This process allows for data linkage across different studies and modalities without revealing the individual’s real identity. Besides DICOM and NifTI, the modern and flexible HL7 FHIR (Fast Healthcare Interoperability Resources) standard enables the interoperability and data exchange across various healthcare domains, including radiology [25,26]. It supports the representation of medical imaging studies and their associated metadata in a structured and standardized manner. HL7 FHIR promotes the use of pseudonymization to maintain patient privacy. By replacing identifying elements with pseudonyms, the data can still be effectively used for research and analysis while preserving the anonymity of the individuals involved.

### 2.2. Data Science Workflow and Data Processing

The data science workflow involves the steps required to train and test machine learning models on decentralized data, as well as pre-process and annotate the collected data. This involves a series of well-defined steps to enable collaborative data analysis while preserving data privacy and security.

Depending on the research question, the required data must be identified and retrieved from the local PACS and HIS (Hospital Information System) first. The pre-processing step may involve data normalization and curation, as well as annotation, segmentation of medical imaging data and complementary medical records. This process should be underlined by a standardized workflow, including templates for structured reports. Researchers collaboratively develop the machine learning model to be used in the federated learning setup. They decide on the architecture, hyperparameters and optimization algorithms that are suitable for the specific research task. The model should be designed to accommodate the distributed nature of the data and take into account the potential heterogeneity of the datasets from different institutions. Models are then implemented and tested locally and sent to the central server to be trained on the decentralized data. The central server coordinates the model updates, while local nodes process data locally without sharing raw data with the central node. The federated learning process begins with local model training at each node using its respective data. The local models are then aggregated on the central server to create a global model that benefits from insights learned from all participating sites. This aggregation is performed in a privacy-preserving manner to ensure that the raw data remain on-site and are not exposed. The global model is evaluated on each local node to assess its performance on diverse datasets. Feedback from participating institutions helps refine the model and improve its generalizability. This iterative process continues until the desired level of model accuracy and performance is achieved. Once the federated learning process is complete, the research findings are interpreted and knowledge gained from the collaborative analysis is shared among all participants. The insights can be used to improve patient care, inform clinical decision making and contribute to medical research advancements.

**Figure 1:**
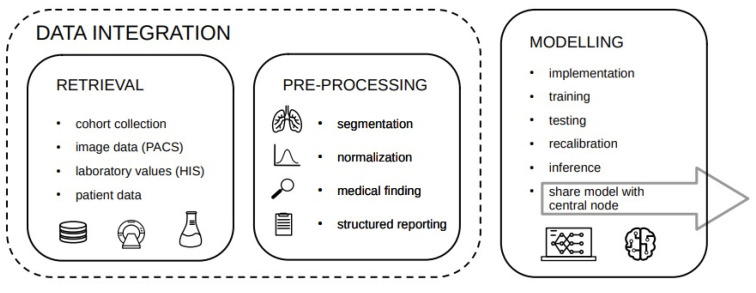
Basic building blocks of the data science workflow.

Since each step requires a subset of tasks which can be rather complex, it is convenient to split the workflow across several services consisting of three major building blocks depicted in Figure 1.

While the choice of the software for data management, finding and annotating mainly relies on the current clinical setup and the tools practitioners and clinicians are used to, we want to highlight a well-suited platform for model related tasks, namely Kaapana. Kaapana is a radiology and radiotherapeutic-focused open-source toolkit for platform provisioning, and comprises federated learning scenarios and AI-based workflows. The data always remain under the authority of the participating site and are processed de-centrally [27].

### 2.3. Local Node Infrastructure Requirements

When working with different services and applications, the requirements on the local node can vary depending on the specific use case and the nature of the services and applications involved.

There must be sufficient hardware resources, including processing power, GPU integration, memory and storage capacity to support the services and applications the local node is running. The specific requirements will depend on the services and applications, but it is important to ensure that the expected workload can be handled without experiencing performance issues.

The network connection should be reliable to support communication with other nodes. This may require configuring firewalls, routers and other network components to allow for the necessary traffic to pass through.

To be secured against unauthorized access and other security threats, implementing access controls, encryption and other security measures can protect sensitive data.

Clinics usually manage their imaging data in a PACS (Picture Archiving and Communication System). PACS communication is necessary when training machine learning models on medical images. Therefore, secure connections and authentication procedures must be established between the PACS and the applied local services.

Compliance with data governance policies and procedures, including those related to data access, data storage and data sharing, requires implementing access controls to ensure that data are handled in accordance with applicable regulations and best practices.

**Figure 2:**
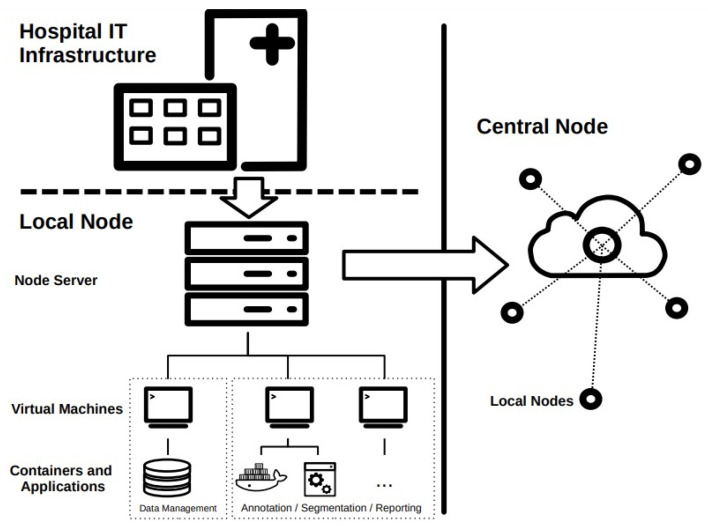
Simplified schematic representation of a possible structure of the local node. The dashed line between the Hospital IT Infrastructure and the Node itself signifies that there might be a separation between the two depending on implementation, as discussed in the subsequent section.

All applications and services utilized in the local node run on virtual machines (VMs) or in containers. Virtualization allows for the creation of virtual environments that simulate hardware and software configurations, enabling different operating systems and applications to run on a single physical machine. This allows for the creation of isolated virtual machines that can be used without interfering with other applications on the host machine. Containerization, on the other hand, allows for the creation of contained environments for applications and their dependencies. Containers provide a way to package an application and its dependencies into a single portable unit, making it easy to move and deploy across different environments. This can be useful in the context of federated learning, as it allows for the creation of standardized environments for running machine learning models on different devices or servers, regardless of their underlying hardware and software configurations. A simplified schematic representation of a possible structure of the local node is depicted in Figure 2.

Figure 2 shows that direct communication only takes place between the local nodes and the central node. In traditional centralized approaches, data from various sources are collected and aggregated on a central server, where a global model is trained. This method provides efficient and straightforward model training, but raises concerns about data privacy, security as sensitive information is pooled together. While the decentralized approach presents challenges such as handling device heterogeneity. However, the promise of preserving privacy while enabling collaborative learning makes decentralized federated learning an increasingly appealing and viable alternative to traditional centralized approaches.

## 3. Results and Discussion

The following section shows experiences and possible issues during the installation of local nodes and analyzes the process of the actual implementation of the local node in the context of the RACOON project at our site.

In order to enable communication between the local node and the central node, it must first be ensured that all data privacy and data security policies are adhered to in this regard. The process of finding an agreement for data sharing was significantly hindered by the differences in regulatory laws of data protection across various jurisdictions. Each country or region has its own set of stringent data protection regulations and local data privacy laws. Differences in data protection laws have led to varying interpretations and definitions of sensitive data, data ownership and responsibilities regarding data breaches. Negotiating data sharing agreements that comply with diverse regulatory laws introduce significant delays in the initiation of research projects. The time and effort required to establish agreements and gain necessary approvals additionally slowed down the whole process. 

Federated learning networks must accommodate various software and hardware setups across participating institutions. Diverse IT infrastructures, data storage systems and imaging modalities can lead to compatibility issues and interoperability challenges. The development of a unified software and hardware framework that can seamlessly integrate with different systems may require substantial effort and expertise.

One of the first steps in setting-up the local node is, to decide where to host the server. This is highly dependent on the local IT infrastructure and varies among sites. The server setup must be sufficiently flexible to accommodate project-related changes in the software architecture, such as updates due to newly implemented functionalities. In addition, the import of data from internal clinical systems such as the hospital information system (HIS), the radiological information system (RIS) and PACS must be guaranteed. Due to the high vulnerability of the data, compliance with local security standards has a very high priority.

Direct integration of the node into the existing hospital IT server landscape can offer several advantages. The necessary interfaces for data transfer should already exist and adherence to security measures requires only a few adjustments, such as the integration of data transfer to the central node and vice versa. Also, backup strategies usually should exist, at least on the server level, including the persistence of the VMs and the host system.

However, those benefits may come at the cost of flexibility. Due to the security policies of the local IT department, direct administrative access to the node can be restricted or not granted at all, thus hampering interaction with the system. Adapting custom project-driven implementations and changes therefore may be accompanied with a high communication effort, resulting in a considerable delay.

Another experienced drawback is the infeasibility of integrating the required hardware specifications. In some cases, contractual obligations prohibit the acquisition of the recommended hardware specifications leading to inconsistencies across sites. In our exemplary case, the existing type of server rack was only capable of integrating a single dedicated GPU. While the pure execution of the services should not be influenced by that, this setup does not scale with applications requiring higher GPU processing power. Besides hardware issues, virtualization can also be a concern when building the node upon an already existing infrastructure. While most sites are in fact using the recommended Hyper-V (Microsoft Corporation, Redmond, WA, USA) hypervisor, there are other solutions utilized at some sites. This incongruence causes conflicts if applications or container images have to be deployed on the virtual machines, exposing dependencies on the VM configuration (e.g., network interface configurations). Those configurations are often provided as Hyper-V-specific configuration files, which are not applicable for other types of hypervisors out of the box. Translating them to the custom configuration format or manually configuring the hypervisor induces an additive time effort and is a source of errors.

The opposite approach of setting up the node directly in the subordinated environment of the radiological clinic gives much more flexibility. At the same time, when hosting the server in a separate environment the responsibility for maintenance, security, communication with the HIS/RIS/PACS and central node, as well as backup strategies must be handled via decentralizing. This is only a possible scenario if communication with the HIS/RIS/PACS is possible at all and in compliance with the security policies of the clinical IT, since opening ports for data transfer means opening access to an external party and exposing vulnerabilities.

Table 1 summarizes the advantages and disadvantages concerning the decision of hosting the server in the existing clinical IT environment vs. setting up the node in the subordinated environment of the radiological department referred to as integrated and separate installation.

The implementation of the local node at our site could not follow the intended standard setting of the project. The server hardware and host system are maintained by the local IT department. Three VMs in total are hypervised via VMware (VMware, Inc., Palo Alto, CA, USA). The corresponding images for deploying the VMs were rolled out in the Hyper-V image format as part of the project. This required conversion to the VMware format at our site. Unfortunately, this process led to unintended delay in several update procedures, because the conversion did not work seamlessly. We did not observe any performance issues concerning communication between workstations in the radiological department and VMs, as well as the data transfer from the PACS to the data management VM. One major benefit of integrating the node in our existing clinical IT infrastructure is the applied backup strategy. Since the VM snapshots are saved in a daily cycle, even in the event of a system failure, there is only minimal data loss.

Based on our experiences, during the process of building the local node, we tried to identify a best-case scenario, comprising flexibility, low maintenance effort and compliance with security obligations. From our perspective, integrating the node should be the preferred way, since building the infrastructure from scratch, including backup-strategies and backup-infrastructure, security precautions and interfaces to the HIS, is associated with high time effort and administrative expense. Nevertheless, if certain specifications cannot be met, installing a separate server can become a more favorable approach. The decision criteria leading to one or the other approach have to be discussed and weighted internally.

The proposed infrastructure in the context of the RACOON project enables viable multicentric research and aggregation of large-scale datasets with a large variety of data sources. In total, the network now consists of 38 local nodes which contributed to the dataset with over 16,000 Thorax CTs and 14,000 curated datasets, including structured reports and segmentations. This shows that this network works efficiently and enables access to high-quality data, empowering future work in radiological and medical research. 

## 4. Conclusions

Federated learning presents a promising alternative to classical machine learning methods in the context of medical and radiology research. Its ability to safeguard data privacy, access diverse datasets and adhere to ethical standards makes it an attractive option for leveraging the potential of machine learning in healthcare. However, challenges related to communication overhead, data heterogeneity and model security must be carefully addressed to fully harness the benefits of federated learning and unlock its transformative potential in the medical field. As the research and development related to federated learning continue to progress, there is promise of ushering collaborative and highly effective medical and radiology applications in the new era of privacy preserving.

Establishing an infrastructure for federated learning and data transfer in the context of a large-scale multi-centric network needs a comprehensive system of local nodes. We have shown basic requirements and building blocks for a local node. Building the local node must comprise flexibility, but also be in concordance with the technical standards of the proposed network structure. We have shown important aspects of the process in building a local node, based on our experience as a participant site of the RACOON project. We pointed out the pitfalls and advantages of an integrated and separate approach to node installation. From our perspective, the building process can benefit from an existing clinical IT infrastructure. However, the exact administrative and technical structure of the sites may vary and thus, the realization remains site-specific. Congruence with other local nodes and the network should thereby be the overarching goal in order to guarantee a functioning interface for data transfer, externally triggered updates and integration. To simplify the implementation, the development of an easily deployable and generic framework should be the focus of future research.

## Figures and Tables

**Table 1:** Advantages and disadvantages of installing the node in the existing clinical IT environment vs. separate installation in local radiology department.

	Integrated Installation	Separate Installation
Flexibility	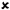 restrictions in virtualization and hardware	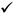 no restrictions
Administrative Access	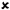 restricted access to VM host 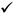 access to VMs	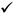 no restrictions
Data Security	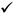 managed externally	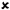 has to be managed separately
Building/Configuration Effort	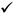 no effort in initial build	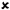 extra space needed 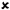 server has to be configured separately
Hardware Integration	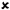 restricted in choice of hardware components 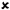 no integration of new components	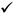 free in choice of components 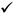 congruence with initial hardware specifications
Maintenance	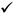 existing backup strategy 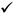 part of the clinical IT maintenance	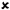 no existing backup strategies 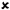 no external maintenance and support 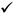 flexibility in setting up custom backups

## Data Availability

No new data were created or analyzed in this study. Data sharing is not applicable to this article.
